# Structural re-positioning, *in silico* molecular modelling, oxidative degradation, and biological screening of linagliptin as adenosine 3 receptor (ADORA3) modulators targeting hepatocellular carcinoma

**DOI:** 10.1080/14756366.2018.1462801

**Published:** 2018-05-16

**Authors:** Bassam M. Ayoub, Yasmeen M. Attia, Mahmoud S. Ahmed

**Affiliations:** a Pharmaceutical Chemistry Department, Faculty of Pharmacy, The British University in Egypt, El-Sherouk City, Egypt;; b The Center for Drug Research and Development (CDRD), Faculty of Pharmacy, The British University in Egypt, El-Sherouk City, Egypt;; c Pharmacology Department, Faculty of Pharmacy, The British University in Egypt, El-Sherouk City, Egypt

**Keywords:** Drug repositioning, adenosine 3 receptor, linagliptin, hepatocellular carcinoma

## Abstract

Chemical entities with structural diversity were introduced as candidates targeting adenosine receptor with different clinical activities, containing 3,7-dihydro-1H-purine-2,6-dione, especially adenosine 3 receptors (ADORA3). Our initial approach started with pharmacophore screening of ADORA3 modulators; to choose linagliptin (LIN), approved anti-diabetic drug as Dipeptidyl peptidase-4 inhibitors, to be studied for its modulating effect towards ADORA3. This was followed by generation, purification, analytical method development, and structural elucidation of oxidative degraded product (DEG). Both of LIN and DEG showed inhibitory profile against hepatocellular carcinoma cell lines with induction of apoptosis at G2/M phase with increase in caspase-3 levels, accompanied by a downregulation in gene and protein expression levels of ADORA3 with a subsequent increase in cAMP. Quantitative in vitro assessment of LIN binding affinity against ADORA3 was also performed to exhibit inhibitory profile at Ki of 37.7 nM. *In silico* molecular modelling showing binding affinity of LIN and DEG towards ADORA3 was conducted.

## Introduction

**Figure 1.**
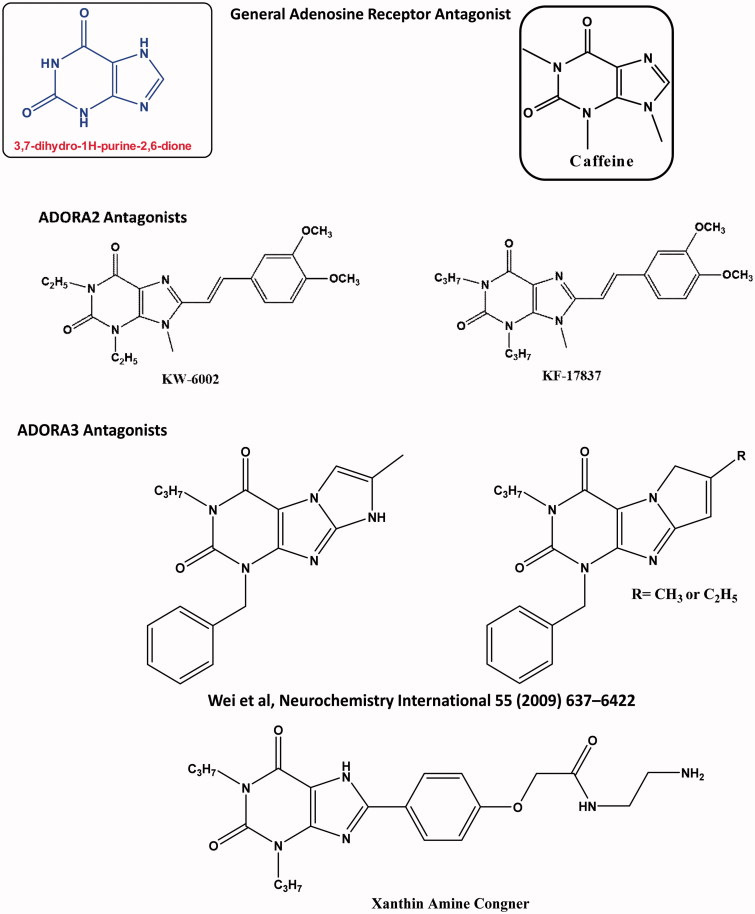
Chemical structures of adenosine receptor modulators with 3,7-dihydro-1H-purine-2,6-dione.

The physiological/pathological mechanism for adenosine has been known *via* activation of four adenosine receptors categorised as ADORA1, ADORA2A, ADORA2B, and ADORA3^1–3^. Adenosine receptor subtypes belong to the seven-transmembrane domain (7TM) receptors or the G protein-coupled receptor (GPCR) family that regulates the main physiological actions by coupling with secondary messenger systems to activate the cellular transduction pathways^4^. Adenosine receptors can be expressed in different tissues with different physiological and pathological roles; where ADORA2A and ADORA2B receptors are coupled to the stimulatory subunit of GPCR (G_s_) to activate adenylate cyclase enzyme to convert the ATP into Cyclic AMP (cAMP), while ADORA1 and ADORA3 receptor binds to the inhibitory subunit of GPCR (G_i_) to inhibit adenylate cyclase to decrease cAMP production^4^^,^^5^. Adenosine receptors have been identified as potential molecular targets for different clinical problems; glaucoma, neurodegeneration, ischemia, cardiac disorders, inflammatory diseases, and cancer, however, FDA only approved Regadenoson ADORA2A selective agonist as a coronary vasodilator for imaging the myocardial perfusion in 2008^6–10^. The modulation of A3 receptor (ADORA3) using small molecules in relation to apoptosis has been controversial, where ADORA3 agonists and antagonists can induce undesirable cytotoxic effect in the cases of neurodegeneration, or desirable cytotoxicity in the cases of cancer^11^^,^^12^. Up till now, there is no FDA approved ADORA3 modulator; this prompted us to follow the drug repurposing approach by assigning the “off-targets” for already approved FDA drugs with established safety profile. On the basis of pharmacophore structural elucidation, chemical compounds containing 3,7-dihydro-1H-purine-2,6-dione have shown potential effect targeting as ADORA2A and ADORA3 modulators, as shown in Figure 1^8^^,^^13–16^.

Linagliptin (LIN) is FDA approved dipeptidyl peptidase-4 (DPP-4) inhibitor as anti-diabetic with 3,7-dihydro-1H-purine-2,6-dione functional group^17^. LIN was selected to be screened for its modulatory activity against ADORA3, this was followed by degradation of LIN to isolate the major oxidative degradation product (DEG)^18^. LIN and DEG were biologically evaluated for their cytotoxicity, modulation/binding affinity to ADORA3, levels of cAMP, and evaluation of apoptosis, followed by validation using *in silico* molecular modelling studies.

## Materials and methods

**Chemicals, reagents, stock solutions, and working solutions:** Pharmaceutical grade LIN certified to contain 99.90% was kindly supplied from Boehringer Ingelheim pharmaceutical company (Ingelheim am Rhein, Germany). All chemicals and HPLC grade solvents were purchased from Sigma-Aldrich (St. Louis, MO). Stock solutions of LIN and DEG (1 mg/ml) were prepared separately in methanol. Working solutions of LIN and DEG (100 μg/ml) were prepared separately in acetonitrile/water (50:50, *v/v*) with appropriate dilutions from stock solutions. All solutions were stored at 4 °C.

**Instrumentation:** LC-MS/MS procedures involved the usage of WATERS ACQUITY UPLC system (S/N F08UPH, Thermo Fisher Scientific, Waltham, MA) using TQ detector (S/N QBA530, Milford, MA) supplemented with ESI source and WATERS ACQUITY UPLC BEH Shield RP C_18_ column (S/N 01563430116023, Fisher Scientific, Dublin, Ireland) with dimensions (150 mm × 2.1 mm, 1.7 μm). The HPLC system was equipped with a Diode Array detector (DAD-3000RS, USA) and an autosampler (WPS-3000TRS, THERMO SCIENTIFIC, Waltham, MA). An Elmasonic S 60 H (Elma Schmidbauer GmbH, Singen, Germany) was used for the degassing of the mobile phases. JENWAY (Stone, UK) digital pH meter was used to adjust and determine the hydrogen ion concentration (pH) of the mobile phase.

### Oxidative degradation of DEG and structural elucidation

**General:** Reaction monitoring was done using TLC plates visualised using UV–vis at wavelength of 254 nm. ^1^H and ^13^C NMR spectra were acquired on a Bruker AVANCE-400 MHz NMR spectrometer (Billerica, MA) in DMSO-d6 using TMS (*δ* = 0 ppm) as the internal standard for ^1^H NMR and CDCl_3_ (*δ* = 77.16 ppm) for ^13^C NMR, with the reporting of coupling constants in Hz and the signal multiplicities are reported as singlet (s), doublet (d), triplet (t), quartet (q), doublet of doublets (dd), doublet of triplets (dt), multiplet (m), or broad (br).

**Degradation of LIN:** DEG was prepared to start with 200 mg of LIN dissolved in 0.5 mM of 30% H_2_O_2_, this was followed by reflux for 1 h. Reaction was monitored by the disappearance of LIN to be quenched by extraction using 3 × 100 ml ethyl acetate/H_2_O mixture. Ethyl acetate layer was concentrated on rotavap to obtain crude yellowish white powder was further purified by silica gel flash column chromatography using 1:1 ethyl acetate/hexane solvent system to yield DEG; 1-((4-methylquinazolin-2-yl)methyl)pyrimidine-2,4,6(1H,3H,5H)-trione (25 mg, 20%). DEG; (1-((4-methylquinazolin-2-yl)methyl)pyrimidine-2,4,6(1H,3H,5H)-trione): **^1^HNMR** (400 MHz, DMSO-d6) *δ* 7.97 (m 1H), 7.90 (m, 1H), 7.72 (m, 2H), 5.05 (s, 2H), 3.11 (s, 2H), 2.91 (s, 3H). ^13^**C NMR** (100 MHz, DMSO-d6) *δ* 175.0, 170.1, 159.0, 157.8, 154.7, 149.3, 134.9, 128.4, 128.2, 126.3, 123.1, 55.2, 44.2, 22.5. M.S. calcd for C_14_H_12_N_4_O_3_, 284.09; found [M + H] 285.05. The spectral data can be shown in Supplementary Figures S1, S2 and S3.

### Analytical method development of LIN and DEG

**HPLC-UV chromatographic conditions:** Concerning HPLC separation using UV detection, it was achieved on a THERMO C_18_ column 100 mm × 2.1 mm (3 μm) applying an isocratic elution based on acetonitrile-phosphate buffer (50:50, *v/v*) at pH 3 as a mobile phase. The photodiode array detector was operated at 226 nm. The mobile phase was filtered through 0.2 μm membrane filter and degassed for 30 min in an ultrasonic bath prior to its use. The mobile phase was pumped through the column at a flow rate of 0.5 ml min^−1^. Column temperature was kept at 25 °C and the injection volume was 10 μL.

**LC-MS/MS chromatographic and mass spectrometric conditions:** The whole procedures were conducted as previously described with slight modification, where the mobile phase was a mixture of 0.1% formic acid and acetonitrile in the ratio of (50:50, *v/v*). The detailed procedures are described in the supporting information.

### Biological screening

***In vitro* assessment of the binding affinity to ADORA3:** Aliquots of 200 μl of 10 ng/ml ADORA3 were prepared. Ten-fold serial dilutions of LIN (10, 1, 0.1, and 0.01 μM) were also prepared. Then, 200 μl of each LIN concentration was mixed with recombinant human ADORA3 (Abcam, Cambridge, MA) and incubated for 10 min. Aliquots of 100 μl of the mixture were then transferred to a plate with anti-ADORA3 antibody (Abcam, Cambridge, MA)-coated wells. A series of 5 standard concentrations (200, 100, 50, 25, and 12.5 pg/ml) of the recombinant ADORA3 were used for the standard curve. Then, 100 μl of 200 pg/ml ADORA3 was used as full activity control wells and incubated for 2 h at 37 °C. The plate was then washed three times and 100 μl of horseradish peroxidase conjugate was then added to the wells and incubated for 1 h at 37 °C. The plate was then washed again for three times and 100 μl of the substrate was added to each well and left in the dark for 30 min followed by 50 μl of stop solution, to be read at 490 nm. Percentage inhibition of each LIN concentration was calculated by dividing the calculated ADORA3 concentration by the full activity control concentration to calculate the IC_50_. The following equation was then used to calculate Ki to reflect on LIN binding affinity to ADORA3:

$$\text{Ki}={\text{IC}}_{50}/(1+([L]/\mathrm{kd}))$$

where [*L*] is the concentration of the substrate used and *Kd* is its dissociation constant.

**Cell lines and culture:** HCC cell lines; HepG2, and Huh7, were obtained from the American Type Culture Collection (ATCC, Manassas, VA). Cells were maintained and cultured as previously described.

**Cytotoxicity assay:** HepG2 and Huh7 cells were seeded, 24 h later application of treatments (LIN, DEG, or DMSO) with 5-serial dilution manner (0.01–100 μM) was conducted to be left for 72 h exposure. Cell viability was expressed in terms of half-maximal inhibitory concentration (IC_50_) based on the absorbance at 570 nm from treated cells versus that from untreated control cells. All experiments were performed in triplicates^19^.

**Experimental design:** HepG2 cells were divided into five groups; (i) the blank control group, (ii) LIN-treated group, (iii) DEG-treated group, (iv) adenosine-treated group, and (v) caffeine-treated group. Groups (iv) and (v) were used in the experiment performed for the determination of ADORA3 protein expression only. All treatments were started 24 h after cells seeding. Gene expression, apoptosis, and cell cycle assays were performed at 48 h treatment exposure, whereas protein expression levels were estimated at 72 h treatment exposure.

**Apoptosis assay using annexin V/propidium iodide staining:** The cells were cultured and treated with LIN or DEG for 48 h, followed by fixation and staining with 5 μl annexin V-FITC and 5 μl propidium iodide (PI) using an Annexin V-FITC Apoptosis Detection Kit (BioVision, Milpitas, CA). FACScan flow cytometer (Beckman Coulter, Brea, CA) was used for analysis, where apoptosis was measured based on the FITC-positive cells.

**Cell cycle analysis:** HepG2 cells were incubated along with either LIN or DEG for 48 h, cells were trypsinised, washed with PBS, and resuspended in cold methanol to be maintained overnight at 4 °C. Collected cells were then resuspended in 250 μl of 1.12% sodium citrate buffer (pH 8.4) together with 12.5 μg RNase, and incubated at 37 °C for 30 min. After centrifugation, cells were resuspended in PBS and filtered. Cell cycle analysis was performed using FACSCalibur Flow Cytometer (BD Biosciences, San Jose, CA).

**Determination of caspase-3 activity:** To determine the effect of LIN and DEG on apoptosis, the active caspase-3 level was measured using human active Caspase-3 ELISA kit (Invitrogen, Carlsbad, CA) according to the manufacturer’s instructions. Optical density was determined at 450 nm.

**Adenosine 3 receptor gene expression analysis using real-time qRT-PCR:** Total RNA was isolated from treated and untreated cells using RNeasy Mini Kit (Qiagen, Mountain View, CA). Complementary DNA (cDNA) synthesis and PCR amplification were carried out using iScript™ One-Step RT-PCR Kit with SYBR^®^ Green (Bio-Rad, Hercules, CA) according to the manufacturer’s instructions using β-actin as the house-keeping gene, as previously described^20^. The PCR primer sequences used in the study and their National Center for Biotechnology Information (NCBI) accession numbers were as follows: ADORA3, forward 5′-ACGG-TGAGGTACCACAGCTTGTG-3′ and reverse 5′-ATACCGCGGGATG GCAGACC-3′ (NCBI accession no.: NM_001302679.1); β-actin, forward 5′-GTCATTCCAAATATGAGATGCGT-3′, and reverse 5′-GCTATCACCTCCCCTGTGTG-3′ (NCBI accession no.: NM_001101.3). Real-time qPCR was performed using a real-time Rotor Gene 3000 PCR cycler (Corbett Research, Sydney, Australia). The relative mRNA expression level was determined using the 2^−ΔΔCt^ analysis method, as previously described^21^.

**Determination of ADORA3 protein levels:** For quantitation of ADORA3 in LIN-, DEG-, adenosine-, and caffeine-HepG2 treated cells, human ADORA3 ELISA Kit (Cube Biosystems, Rokville, MD) was used according to the manufacturer’s instructions. The optical density was determined within 30 min using a microplate reader set at 450 nm.

Cyclic AMP assay: In order to determine the intracellular cAMP levels in cells treated with either LIN or DEG, cAMP ELISA kit (Cloud-Clone Corp., Katy, TX) was used according to the manufacturer’s instructions. This assay uses a competitive inhibition enzyme immunoassay technique for the *in vitro* quantitative estimation of cAMP. After performing all steps of the assay, the optical density was determined at 450 nm. The intensity of the colour developed is reverse proportional to the concentration of cAMP in the sample.

**Statistical analysis:** All values are presented as means ± standard deviation (SD). Statistical analysis was performed by one-way analysis of variance (One-way ANOVA) followed by Bonferroni *post hoc* test for multiple comparisons whereas the relative gene expression data analysis was performed by Student’s *t*-test for independent samples using GraphPad Prism (GraphPad Software, La Jolla, CA). Statistical significance was determined at *p* < .05.

### *In silico* molecular modelling simulations

**Molecular docking studies:** LIN and DEG were designed, prepared and energy minimised using MMFF94, followed by multi conformers generation using OMEGA, to be docked along with the energy minimised ADORA3 receptor (PDB ID: 1OEA) using FRED, as previously reported^22^^,^^23^.

## Results and discussion

**Figure 2.**
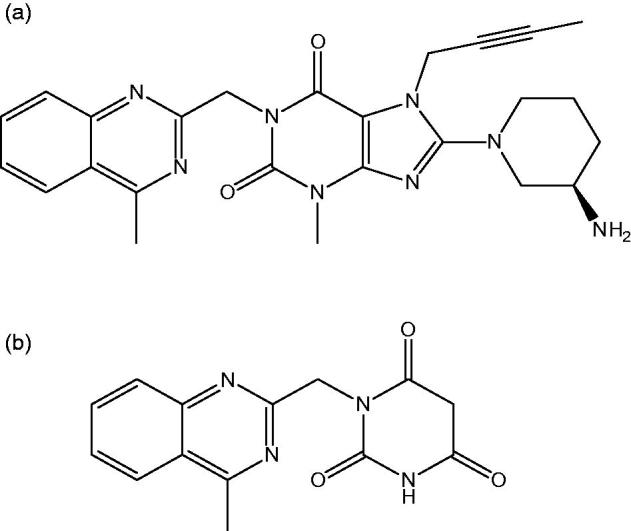
Two dimensional structure for (a) Linagliptin (b) Proposed chemical structure of linagliptin degradation product (DEG).

**Identification and structure elucidation of LIN major degradation product:** Lingaliptin was exposed to oxidation conditions in the presence of peroxides, followed by purification using flash column chromatography to yield pure compound. Spectroscopic techniques were employed to elucidate the structure of degraded product using IR, ^1^H and ^13^C NMR to reveal that DEG; 1-((4-methylquinazolin-2-yl)methyl)pyrimidine-2,4,6(1H,3H,5H)-trione was obtained at 20% yield, as shown in Figure 2.

**Figure 3.**
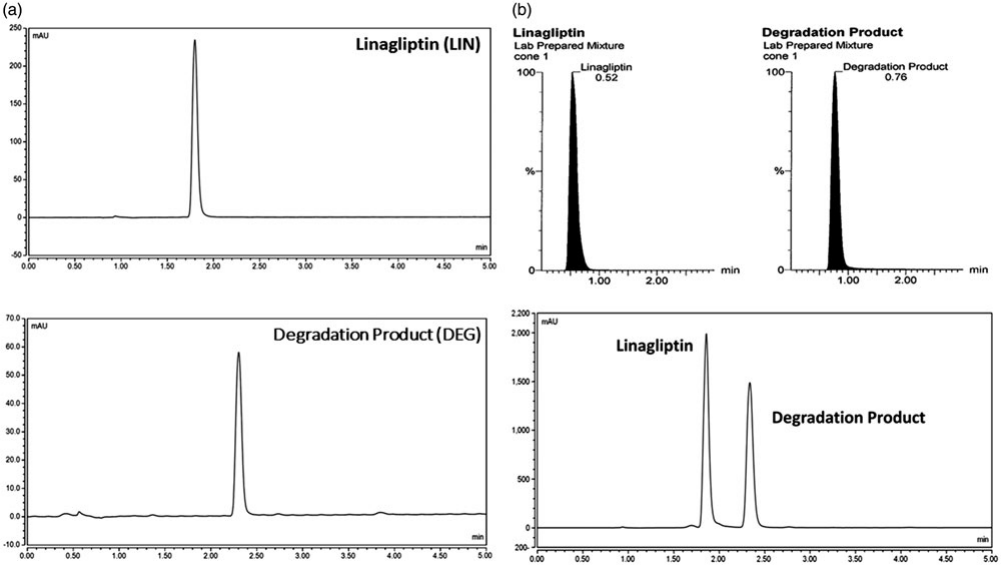
HPLC chromatograms of (a) Linagliptin (10 µg mL^−1^) and the proposed degradation product (2 µg mL^−1^). (b) Multiple reaction monitoring (MRM) chromatogram (a) of linagliptin (*m/z* = 473.11–420.07) and the degradation product (*m/z* = 285.05–156.93) and HPLC chromatogram (b) of a lab prepared mixture of linagliptin (80 µg mL^−1^) and the degradation product (50 µg mL^−1^).

NMR data showed the catalytic cleavage of LIN leaving the 4-methyl quinazoline moiety and opening the purine 2,6 Dione moiety to obtain pyrimidine-2, 4, 6 (1H, 3H, 5H)-trione moiety, where ^13^C DEPT135 showed the presence of 1 –CH3, 2 –CH2, 4 –CH groups. This was validated with 3D-Scan results (Supplementary Figure S4), showing the quinazoline moiety. This was followed by simultaneous determination of LIN and DEG with high sensitivity according to their using HPLC-DAD at 226 nm, as shown in Figure 3(a). The mobile phase was adjusted to be in the acidic region (2.5 and 3.5) to ensure its value below the pka of LIN by more than two units. Moreover, MRM chromatogram and HPLC chromatogram of a laboratory prepared mixture of LIN and DEG, as shown in Figure 3(b).

**Figure 4.**
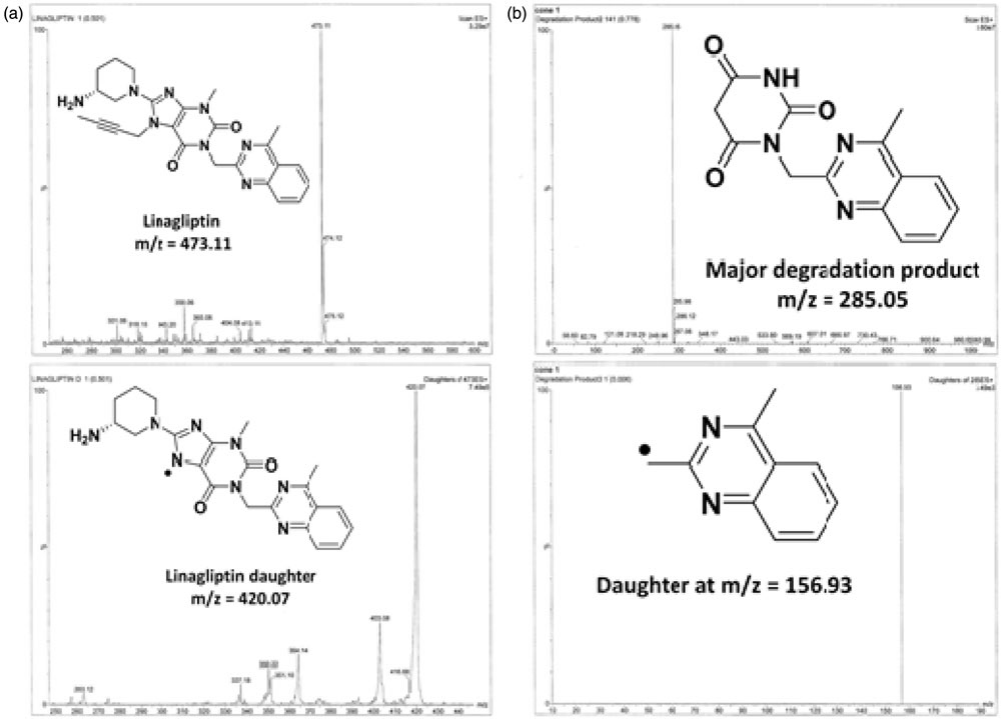
Full scan mass spectrum and daughter ion mass spectrum in positive ESI ion detection mode with the proposed fragment. (a) Linagliptin and (b) linagliptin major degradation product (DEG).

Meanwhile, a simple LC-ESI-MS/MS method was used to detect the molecular weight of DEG, where both LIN and DEG fragments can be explained, as shown in Figure 4. The protonated molecular ions [M + H]^+^ of 473.11 for LIN and 285.05 for DEG were observed on the full scan mass spectra. The collision energy has resulted in the production of characteristic ions. Upon utilisation of sufficient collision activated dissociation gas and collision energy, the following MS/MS transitions were carefully chosen, 473.11 → 420.07 and 285.05 → 156.93 for LIN and DEG, respectively.

Although few methods were reported in the literature as stability indicating assays for LIN^24^, it is worth mentioning that separation and/or characterisation and biological evaluation of the major degradation product strategy was not previously investigated. Furthermore, the new developed LC-MS/MS method enabled the maximum sensitivity (LOQ of 40 ng/ml) than the reported HPLC methods^24–26^ to ensure the absence of any degradation products in the marketed formulation. The first stability indicating LC-MS/MS method was developed for sensitive determination of LIN and its major oxidative degradation product. Best linearity results were obtained in the range of (40–800) ng/ml with correlation coefficient of 0.9995. Accuracy of the results was ensured by good recovery percent of three different concentrations (400, 450, and 550 ng/ml) as 101.60% ± 1.81. Precision was confirmed based on %R.SD of 1.78. Application on the marketed dosage form, TRAJENTA tablets (5 mg), was achieved successfully and resulted in acceptable recovery of 97.74% with no detection for degradation product in the tablets dosage form. It is worth noting that the degradation entity possessing pyrimidine-2,4,6 (1H, 3H, 5H)-trione structural moiety has been previously reported as a degraded product/s in other gliptins such as alogliptin and trelagliptin^27^.

***In vitro* assessment of LIN binding affinity to ADORA3:** The results of the *in vitro* binding assay showed strong binding affinity of LIN to ADORA3 with a Ki of 37.7 nM at IC_50_ of 0.5 μM.

**Figure 5.**
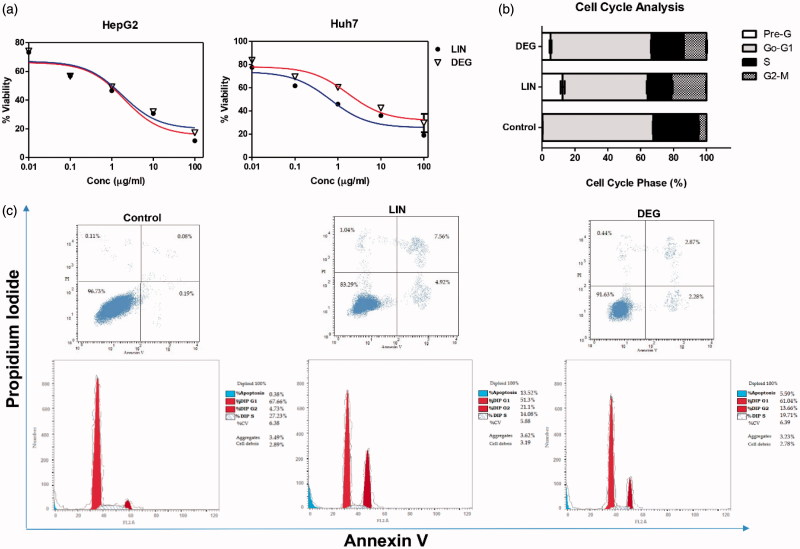
Effect of linagliptin and its major oxidative degradation product on viability in HepG2 and Huh7 cell lines and cell cycle progression in HepG2 cell line. (a) Dose-response plots of linagliptin (LIN) and its degradation product (DEG) on HepG2 and Huh7 cell lines after 72 h exposure, as detected by MTT assay. (b,c) DNA content-based cell cycle analysis in HepG2 cells treated with either LIN or DEG.

***In vitro* cytotoxicity and cell cycle analysis of LIN and DEG:** MTT assay showed that both LIN and DEG decreased the rate of cell proliferation in both HepG2 and Huh7 cell lines, as compared to the corresponding control. The IC_50_ of LIN and DEG on HepG2 cell line was found to be 1.97 and 1.79 μM, respectively. Moreover, the IC_50_ determined on Huh7 cell line was found to be 0.67 and 1.72 μM for LIN and DEG, respectively. As for the cell cycle analysis, as shown in Figure 5(a), LIN and DEG caused cell cycle arrest at G2/M phase where a 4.4- and 2.87-fold increase (*p* < .0001) in the number of HepG2 cells at this phase was observed, respectively, as shown in Figure 5(b). Interestingly, LIN and DEG caused also a 46.37- and 19.35-fold increase (*p* < .0001) in the percentage of HepG2 cells in the pre-apoptotic phase, respectively.

To further assess the effect of LIN and DEG on apoptosis, active caspase-3 protein levels were investigated in treated cells. LIN and DEG were found to cause a 9.62- and 6.5-fold increase (*p* < .0001) in caspase-3 protein levels, as compared to control, respectively.

There is a growing body of evidence suggesting a potential role of ADORA3 in tumourigenesis, where it was found to be upregulated in different tumour types^28–30^ including HCC^12^. Moreover, ADORA3 partial agonists were previously described to cause cell cycle arrest where pro- and anti-apoptotic effects have been reported^31^. This was explained in light of the ability of such ligands to suppress the transcription of the cell cycle key regulatory genes such as cyclin D_1_ and c-Myc^12^_._ Increased levels of caspase-3 were also previously observed in HCC tumour-bearing rats treated with the ADORA3 agonist, CF102^12^.

**Figure 6.**
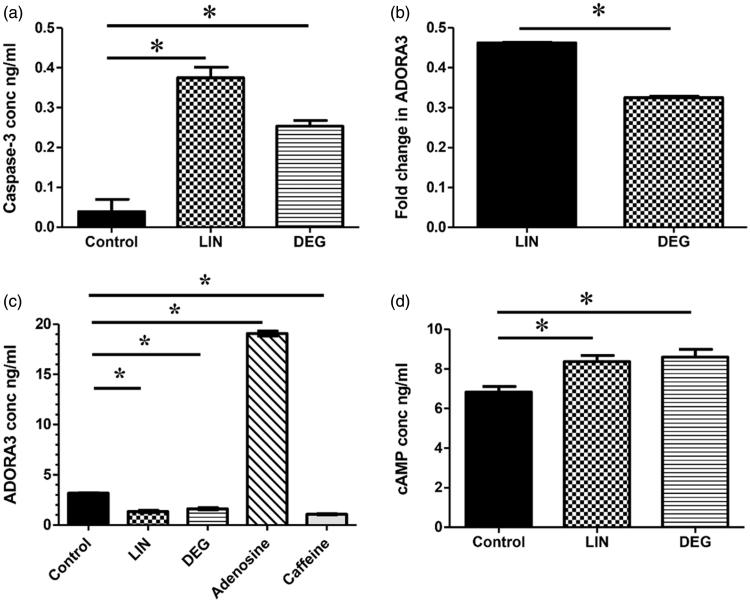
Effect of linagliptin and its major oxidative degradation product on caspase-3, ADORA3, and intracellular cAMP levels. (a) Active caspase-3 protein levels in control, linagliptin (LIN)-treated, and the degradation product (DEG)-treated HepG2 cells. (b) ADORA3 relative mRNA expression levels in LIN- and DEG-treated HepG2 cells. (c) ADORA3 protein expression levels in LIN-, DEG-, adenosine-, & caffeine-treated HepG2 cells. (d) Intracellular cAMP protein levels in control, LIN-, and DEG-treated HepG2 cells. Gene expression levels were estimated using real-time qPCR. Relative mRNA expression level (fold change) was determined using the 2^−ΔΔCt^ analysis method. Protein levels were estimated using ELISA. Values are presented as means ± SD Statistical analysis was performed using one-way analysis of variance (One-way ANOVA) followed by Bonferroni *post hoc* test while Student’s *t-*test was used for relative mRNA data analysis. *Significantly different (at *p* < .05) versus control untreated cells (a, c, and d) or LIN-treated group (b).

**Effect of LIN and DEG on gene and protein levels of ADORA3 and intracellular cAMP:** To investigate the effect of LIN and DEG on ADORA3, both gene and protein levels were estimated in treated cells. As shown in Figure 6, LIN caused a decrease in ADORA3 relative mRNA expression level by 53.8% (*p* < .0001) whereas DEG caused a 67.49% (*p* < .0001) downregulation in the aforementioned gene, as compared to control. Additionally, LIN showed a 57.46% decrease in the ADORA3 protein levels while a 49% decrease was reported in DEG-treated cells, as compared to control. Moreover, in order to enhance our understanding of the modulatory effect of LIN on ADORA3, the protein expression pattern obtained by LIN was compared to that obtained by caffeine, as an antagonist for ADORA3, and adenosine, as an agonist for the same receptor. Furthermore, to demonstrate the presence of functional ADORA3 and investigate whether changes in receptor gene and protein expression were reflected at a functional level, cAMP levels were measured. As shown in Figure 6, LIN and DEG caused a 1.23- and 1.26-fold increase (*p* = .0012) in cAMP levels in HepG2 cells, as compared to untreated control cells.

These results suggested that LIN and DEG could have probably exerted their antiproliferative effect on HCC cells through downregulation of ADORA3 that are overly expressed in these cells, as mentioned earlier. Desensitisation of ADORA3, as part of the GPCR superfamily, after exposure to agonist stimulation, has been extensively studied^32^. Both homologous and heterologous desensitisation mechanisms, resulting from receptor phosphorylation and hence uncoupling from its G protein, were proposed^33^. Interestingly, these results corroborated with the levels of cAMP in treated cells showing that signal transduction initiated after ADORA3 stimulation was desensitised as well. Signal termination following receptor desensitisation despite continued exposure to the agonist was previously reported^34^. However, LIN modulatory pattern for ADORA3 was more similar to that observed with caffeine (6-fold increased expression) rather than adenosine (4-fold decreased expression). Meanwhile, the reported desensitisation with GPCRs in general and adenosine receptors in particular, as explained above, may controvert this claim. Accordingly, in order to confirm that this modulation was due to desensitisation rather than antagonistic activity, additional studies are recommended to further explore the nature of this ligand-receptor complex interaction.

**Figure 7.**
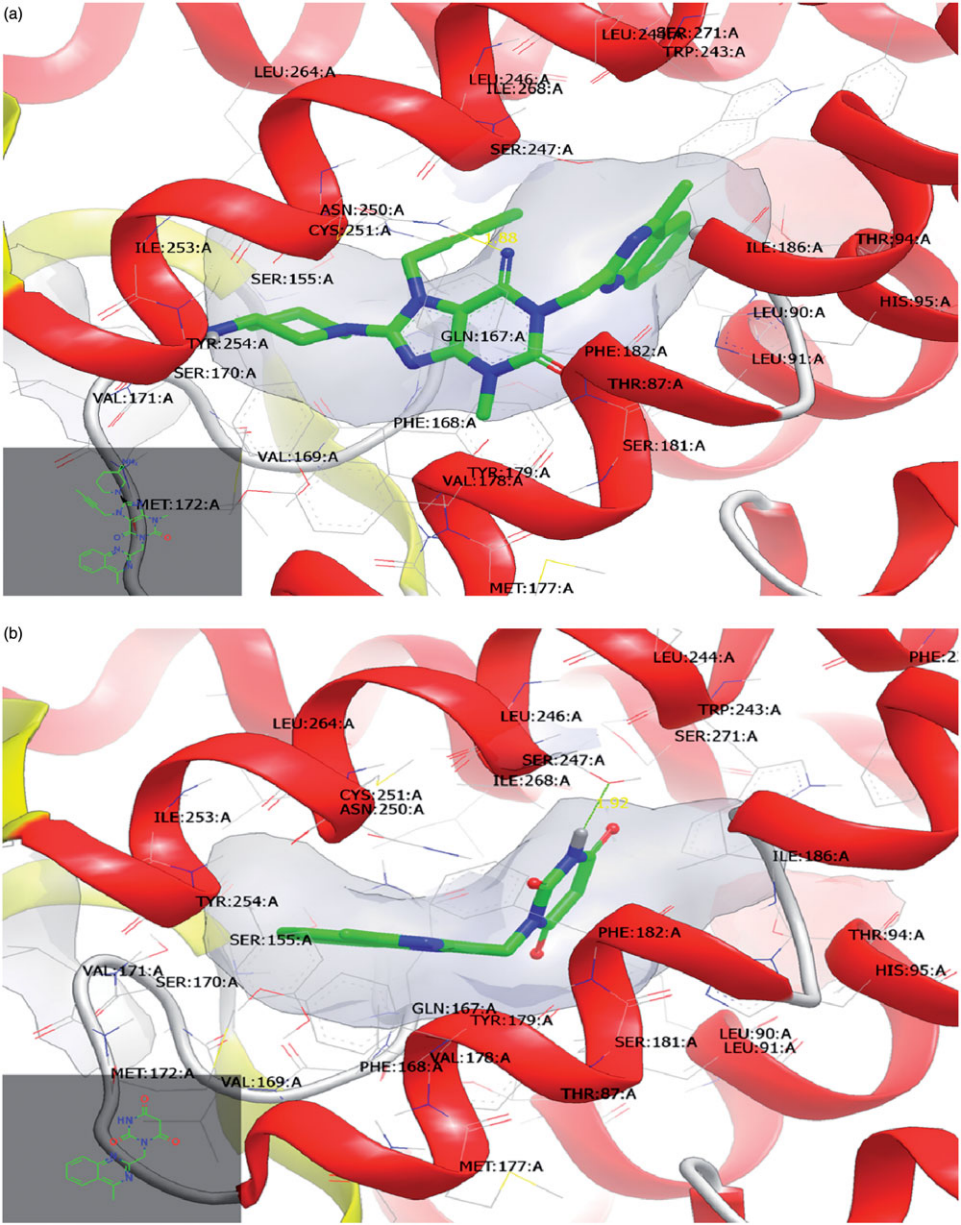
Visual representation of ADORA3 crystal structure. (a) Lingaliptin is showing hydrophobic–hydrophobic interactions along with the receptor surface and one hydrogen bond along with ASN:250:A. (c) Degradation Product (DEG) is showing potential hydrophobic–hydrophobic interactions along with the receptor surface and one hydrogen bond along with SER:247:A.

***In silico* molecular modelling:** The x-ray crystal structure for ADORA3 has not been resolved up till now, that is why ADORA2A crystal structures have been used templates due to their high sequence alignment similarity based on PDB ID:3EML to be used for docking execution using Openeye molecular modelling software, to show potential binding affinity of LIN *via* hydrophobic–hydrophobic interaction along with the previously reported modulators along with ADORA3, л–л interactions between the 2,6 purine dione skeleton along with Phe:182:A and TYR:254:A and hydrogen bond interaction along with ASN:250:A at 1.88 A°, as shown in Figure 7(a). DEG showed potential hydrophobic–hydrophobic interactions within the whole receptor pocket, in addition to hydrogen bond interaction along with SER:247:A at 1.92 A°, as shown in Figure 7(b).

## Conclusions

Investigation of 3,7-dihydro-1H-purine-2,6-dione structural moiety as a potential scaffold to target adenosine 3 receptors (ADORA3) modulator towards treatment of hepatocellular carcinoma directed us to re-purpose LIN, already FDA approved anti-diabetic drug with established safety profile, as adenosine 3 receptors (ADORA3) modulator targeting hepatocellular carcinoma, in addition to its oxidative product. This was further validated by the ability of LIN and its oxidative product to induce cell cycle arrest at G2/M phase with an increase in the apoptotic active caspase-3 protein levels. This study offered novel scaffold that can be further investigated at the structural level to offer novel chemical entities targeting ADORA3.

## Supplementary Material

IENZ_1462801_Supplementary_Material.pdf

## References

[1] Linden J. Molecular approach to adenosine receptors: receptor-mediated mechanisms of tissue protection. Annu Rev Pharmacol Toxicol 2001;41:775–87. 11264476 10.1146/annurev.pharmtox.41.1.775

[2] Fredholm B, Ijzerman A, Jacobson K, et al International Union of Pharmacology. XXV. Nomenclature and classification of adenosine receptors. Pharmacol Rev 2001;53:527–52. 11734617 PMC9389454

[3] Sadana R, Dessauer C. Physiological roles for G protein-regulated adenylyl cyclase isoforms: insights from knockout and overexpression studies. Neurosignals 2009;17:5–22. 18948702 10.1159/000166277 PMC2790773

[4] Tang X, Wang Y, Li D, et al Orphan G protein-coupled receptors (GPCRs): biological functions and potential drug targets. Acta Pharmacologica Sinica 2012;33:363–71. 22367282 10.1038/aps.2011.210 PMC4077139

[5] Pierce K, Premont R, Lefkowitz R. Seven-transmembrane receptors. Nat Rev Mol Cell Biol 2002;3:639–50. 12209124 10.1038/nrm908

[6] Jacobson K, In adenosine receptors in health and disease. Berlin Heidelberg: Springer; 2009 1–24 p.

[7] Chen J, Eltzschig H, Fredholm B. Adenosine receptors as drug targets-what are the challenges? Nat Rev Drug Discov 2013;12:265–86. 23535933 10.1038/nrd3955 PMC3930074

[8] Müller C, Jacobson K. Recent developments in adenosine receptor ligands and their potential as novel drugs. Biochim Et Biophys Acta 2011;1808:1290–308. 10.1016/j.bbamem.2010.12.017 PMC3437328 21185259

[9] Mustafa S, Morrison R, Teng B, Pelleg A. Adenosine receptors and the heart: role in regulation of coronary blood flow and cardiac electrophysiology. Handb Exp Pharmacol 2009;161–88. 19639282 10.1007/978-3-540-89615-9_6 PMC2913612

[10] Thomas G, Thompson R, Miyamoto M, et al The RegEx trial: a randomized, double-blind, placebo- and active-controlled pilot study combining regadenoson, a selective A (2A) adenosine agonist, with low-level exercise, in patients undergoing myocardial perfusion imaging. J Nucl Cardiol 2009;16:63–72. 19152130 10.1007/s12350-008-9001-9

[11] Fishman P, Bar-Yehuda S, Liang B, Jacobson K. Pharmacological and therapeutic effects of A_3_ adenosine receptor (A_3_AR) agonists. Drug Discovery Today 2012;17:359–66. 22033198 10.1016/j.drudis.2011.10.007 PMC3289754

[12] Bar-Yehuda S, Stemmer S, Madi L, et al The A3 adenosine receptor agonist CF102 induces apoptosis of hepatocellular carcinoma via de-regulation of the Wnt and NF-kappaB signal transduction pathways. Int J Oncol 2008;33:287–95. 18636149

[13] Ciancetta A, Jacobson K. Structural probing and molecular modeling of the A_3_ adenosine receptor: a focus on agonist binding. Molecules 2017;22:449. 10.3390/molecules22030449 PMC5471610 28287473

[14] Tosh D, Deflorian F, Phan K, et al Structure-guided design of A_3_ adenosine receptor-selective nucleosides: combination of 2-arylethynyl and bicyclo[3.1.0]hexane substitutions. J Med Chem 2012;55:4847–60. 22559880 10.1021/jm300396n PMC3371665

[15] Deganutti G, Cuzzolin A, Ciancetta A, Moro S. Understanding allosteric interactions in G protein-coupled receptors using Supervised Molecular Dynamics: a prototype study analysing the human A_3_ adenosine receptor positive allosteric modulator LUF6000. Bioorg Med Chem 2015;23:4065–71. 25868747 10.1016/j.bmc.2015.03.039

[16] (A) Yaziji V, Rodríguez D, Gutiérrez-de-Terán H, et al Pyrimidine derivatives as potent and selective A_3_ adenosine receptor antagonists. J Med Chem 2011;54:457–71. (B) Wei J, Li H, Qu W, Gao Q. Molecular docking study of A(3) adenosine receptor antagonists and pharmacophore-based drug design. Neurochem Int 2009;55:637–42. 10.1021/jm100843z 21186795

[17] Forst T, Pfützner A. Linagliptin, a dipeptidyl peptidase-4 inhibitor with a unique pharmacological profile, and efficacy in a broad range of patients with type 2 diabetes. Expert Opin Pharmacother 2012;13:101–10. 22149370 10.1517/14656566.2012.642863

[18] Mourad S, El-Kimary E, Hamdy D, Barary MA. Stability-indicating HPLC-DAD method for the determination of linagliptin in tablet dosage form: application to degradation kinetics. J Chromatogr Sci 2016;54:1560–6. 10.1093/chromsci/bmw103 27334290

[19] Mosmann T. Rapid colorimetric assay for cellular growth and survival: application to proliferation and cytotoxicity assays. J immunol Methods 1983;65:55–63. 6606682 10.1016/0022-1759(83)90303-4

[20] Grove S, Faller R, Soleim K, Dannevig B. Absolute quantitation of RNA by a competitive real-time RT-PCR method using piscine nodavirus as a model. J Virol Methods 2006;132:104–12. 16229902 10.1016/j.jviromet.2005.08.022

[21] Livak K, Schmittgen T Analysis of relative gene expression data using real-time quantitative PCR and the 2(-Delta Delta C(T)) Method)). Methods 2001;25:402–8. 11846609 10.1006/meth.2001.1262

[22] (A) Katritch V, Kufareva I, Abagyan R. Structure based prediction of subtype-selectivity for adenosine receptor antagonists. Neuropharmacology 2011;60:108–15. (B) Floris M, Sabbadin D, Medda R, et al Adenosiland: walking through adenosine receptors landscape. Eur J Med Chem 2012;58:248–57. 10.1016/j.neuropharm.2010.07.009 PMC2980563 20637786

[23] (A) Ahmed M, Kopel L, Halaweish F. Structural optimization and biological screening of a steroidal scaffold possessing cucurbitacin‐like functionalities as B‐raf inhibitors. Chem Med Chem 2014;9:1361–7. (B) Alsayari A, Kopel L, Ahmed M, et al Design, synthesis, and biological evaluation of steroidal analogs as estrogenic/anti-estrogenic agents. Steroids 2017;118:32–40. (C) Hawkins P, Skillman A, Warren G, et al Conformer generation with OMEGA: algorithm and validation using high quality structures from the protein databank and cambridge structural database. J Chem Inf Model 2010;50:572–84. (D) Mcgann M. FRED pose prediction and virtual screening accuracy. J Chem Inf Model 2011;5:578–96.

[24] Zhang H, Sun L, Zou L, et al Identification, characterization and HPLC quantification of process-related impurities in Trelagliptin succinate bulk drug: six identified as new compounds. J Pharm Biomed Anal 2016;128:18–27. 27209451 10.1016/j.jpba.2016.04.041

[25] Deng S, Li Z, Jiang J, et al Determination of related substances in trelagliptin succinate by RP-HPLC and identification of impurities from acid degradation by LC-MS/MS. Chin J New Drugs 2016;25:226–31.

[26] Zhou Y, Zhou W, Sun L, et al Characterization of process-related impurities including forced degradation products of alogliptin benzoate and the development of the corresponding reversed-phase high-performance liquid chromatography method. J Sep Sci 2014;7:1248–55. 10.1002/jssc.201301384 24616424

[27] Zaghary W, Mowaka S, Hassan M, Ayoub B. Suitability of various chromatographic and spectroscopic techniques for analysis and kinetic degradation study of trelagliptin. Sci Rep 2017;7:17255. 29222475 10.1038/s41598-017-17642-1 PMC5722889

[28] Fishman P, Bar-Yehuda S, Ardon E, et al Targeting the A3 adenosine receptor for cancer therapy: inhibition of prostate carcinoma cell growth by A3AR agonist. Anticancer Res 2003;23:2077–83. 12894581

[29] Madi L, Bar-Yehuda S, Barer F, et al A3 adenosine receptor activation in melanoma cells: association between receptor fate and tumor growth inhibition. J Biol Chem 2003;278:42121–30. 12865431 10.1074/jbc.M301243200

[30] Ohana G, Bar-Yehuda S, Arich A, et al Inhibition of primary colon carcinoma growth and liver metastasis by the A3 adenosine receptor agonist CF101. Br J Cancer 2003;89:552–8. 14562031 10.1038/sj.bjc.6601315 PMC2394357

[31] Aghaei M, Panjehpour M, Karami-Tehrani F, Salami S. Molecular mechanisms of A3 adenosine receptor-induced G1 cell cycle arrest and apoptosis in androgen-dependent and independent prostate cancer cell lines: involvement of intrinsic pathway. J Cancer Res Clin Oncol 2011;137:1511–23. 21830157 10.1007/s00432-011-1031-z PMC11828242

[32] Xiao Z, Yao Y, Long Y, Devreotes P. Desensitization of G-protein-coupled Receptors Agonist-Induced phosphorylation of the chemiattractant receptorcAR1 lowers its intrinsic affinity for cAMP. J Biol Chem 1999;274:1440–8. 9880518 10.1074/jbc.274.3.1440

[33] Ferguson S. Evolving concepts in G protein-coupled receptor endocytosis: the role in receptor desensitization and signaling. Pharmacol Rev 2001;53:1–24. 11171937

[34] Trincavelli M, Tuscano D, Cecchetti P, et al Agonist-induced internalization and recycling of the human A(3) adenosine receptors: role in receptor desensitization and resensitization. J Neurochem 2000;75:1493–501. 10987829 10.1046/j.1471-4159.2000.0751493.x

