# Supplementary material for: Structural re-positioning, in silico molecular modelling, oxidative degradation, and biological screening of linagliptin as adenosine 3 receptor (ADORA3) modulators targeting hepatocellular carcinoma
Source: J Enzyme Inhib Med Chem. 2018 May 16;33(1):858–66. doi: 10.1080/14756366.2018.1462801 (PMC6010121; doi:10.1080/14756366.2018.1462801)
Supplement: IENZ_1462801_Supplementary_Material.pdf [file IENZ_A_1462801_SM3582.pdf]

Structural repositioning, *in-silico* molecular modeling, oxidative degradation, and biological screening of linagliptin as adenosine 3 receptor (ADORA3) modulators targeting hepatocellular carcinoma

Bassam M. Ayoub [a, b, ‖], Yasmeen M. Attia [b, c, ‖], Mahmoud S. Ahmed [a, b, ‖*]
* Corresponding author: Mahmoud S. Ahmed, Ph.D.
Pharmaceutical Chemistry Department
The Center for Drug Research and Development (CDRD)
Faculty of Pharmacy, The British University in Egypt
Suez Road, El Sherouk City, Cairo Governorate 11837
mahmoud.salama@bue.edu.eg

Supporting Data

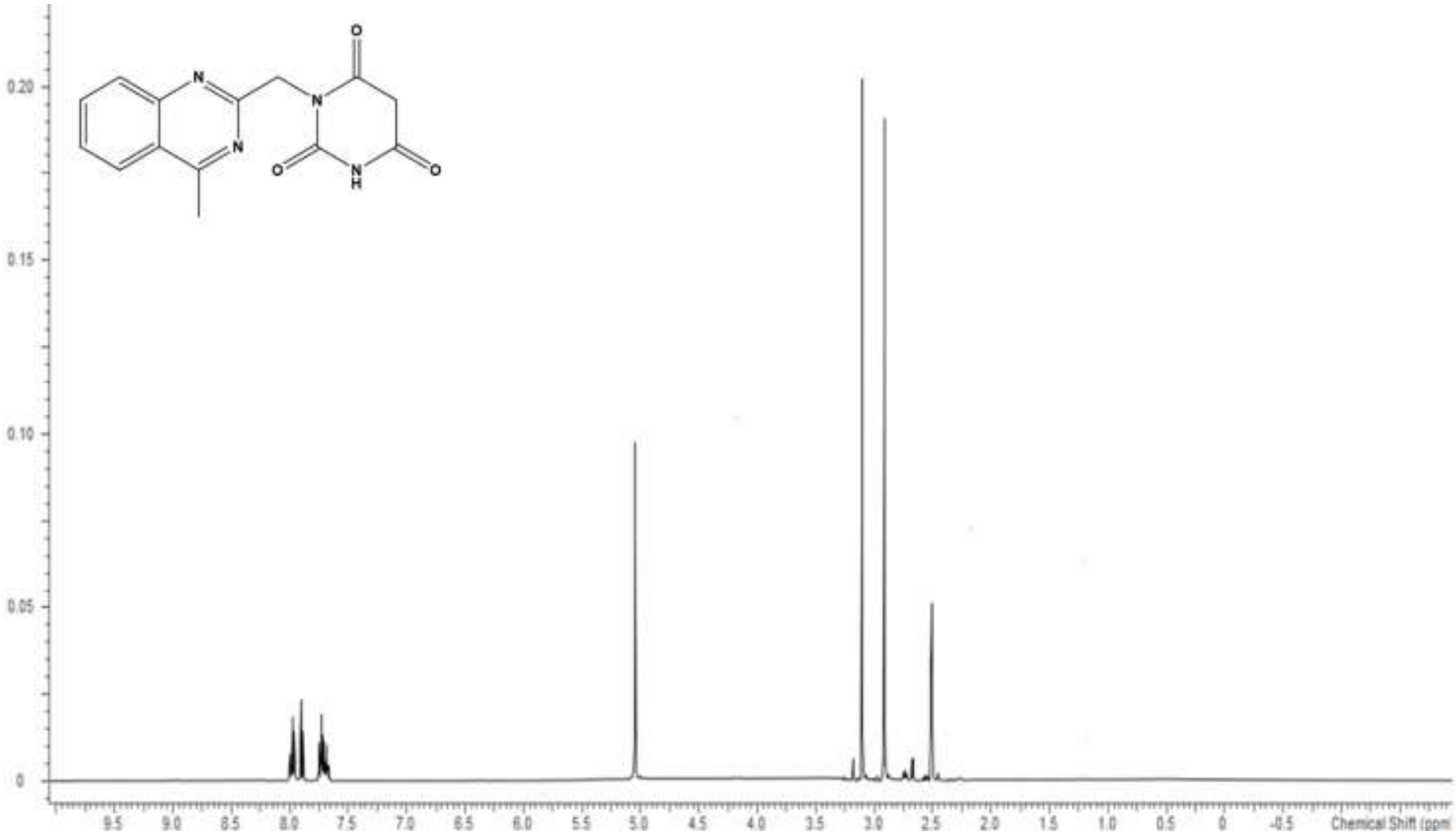

Figure S1: ^{1}HNMR spectra for Degradation Product (DEG)

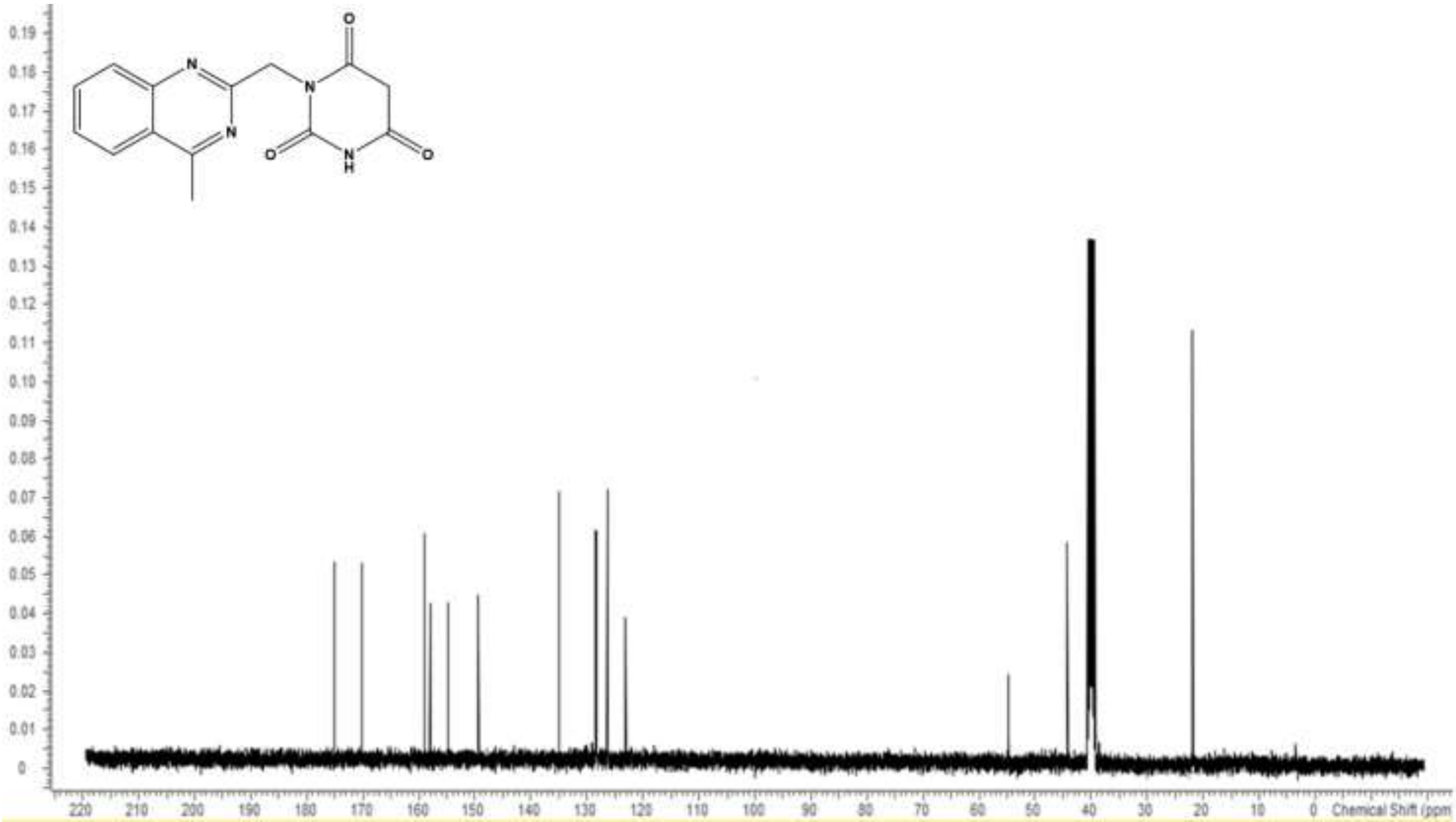

Figure S2: C^{13}NMR spectra for Degradation Product (DEG)

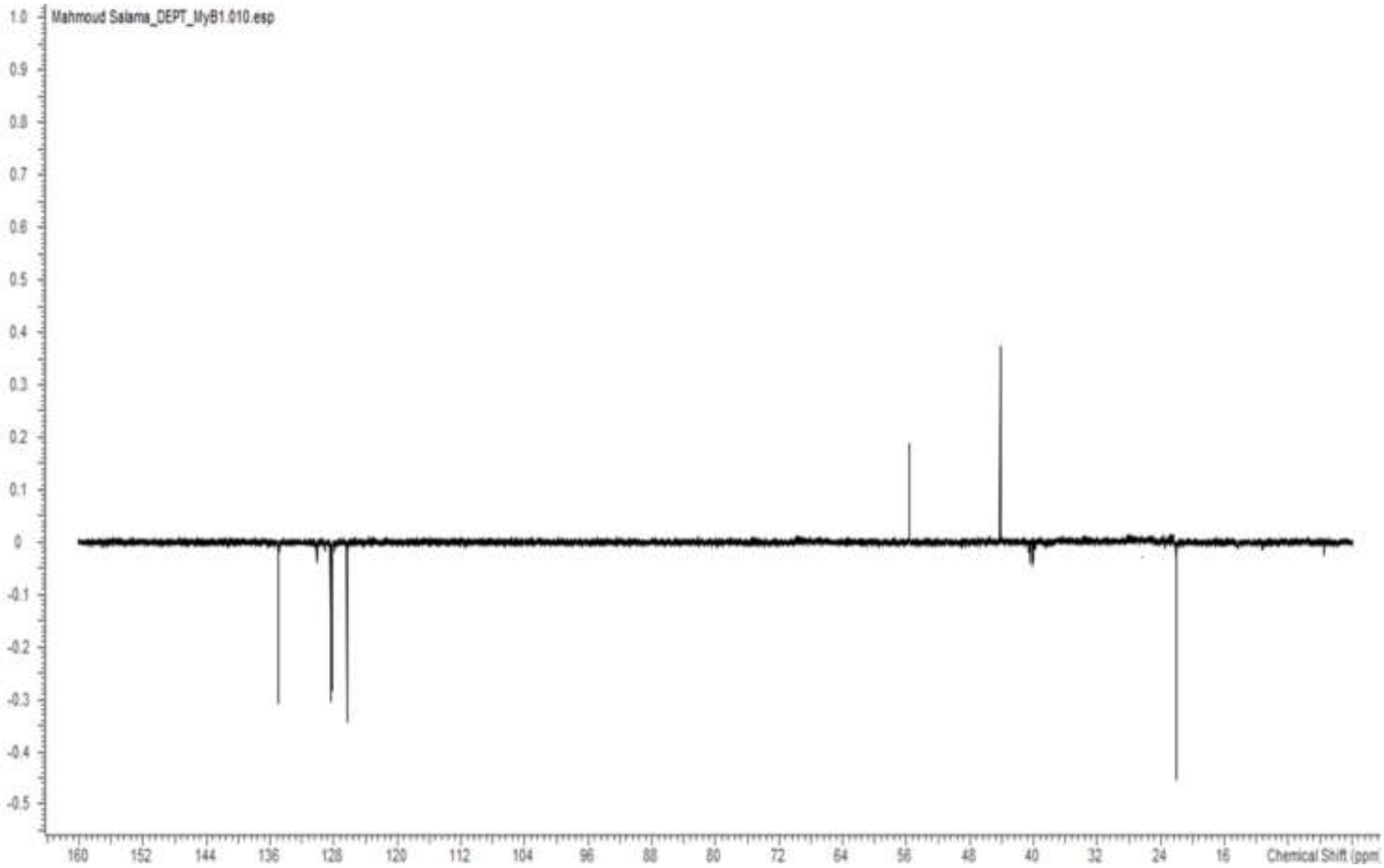

Figure S3: DEPT135 spectra for Degradation Product (DEG)

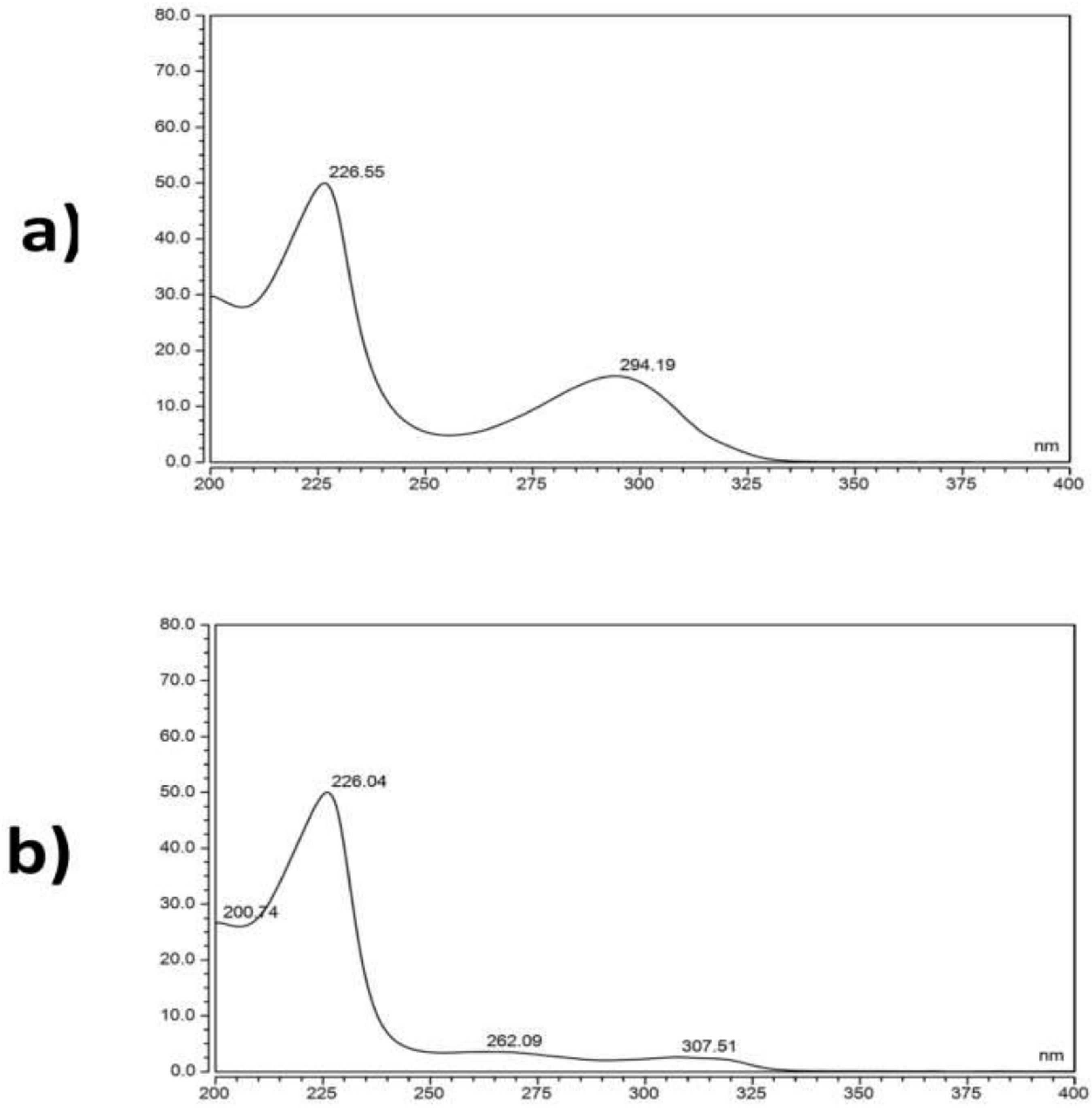

Figure S4: 3D spectrum scan of (a) Linagliptin and (b) the proposed degradation product.

LC-MS/MS chromatographic and mass spectrometric conditions:
The whole The column temperature was kept at 25°C, the injection volume used was 10 µL, and the flow rate was 0.3 mL/min with 3 min as the run time. Cone voltage was set at 30 V; source temperature was set at 150°C, and the collision energy was set at 30 eV for both drugs to enable multiple reaction monitoring (MRM) of the transition pairs

of m/z 473.11 to 420.07 for LIN and m/z 285.05 to 156.93 for DEG in the positive mode utilizing Electro Spray Ionization (ESI). The following parameters were applied: turbo ions spray at 400°C, capillary temperature at 275°C, sheath and auxiliary gas at 15 and 2 psi, respectively, ion spray voltage of 3800 V, capillary voltage of 4 KV, capillary offset of 35 and de-solvating line temperature at 400°C.
